# Photoinduced Radical Persistent Luminescence in Semialiphatic Polyimide System with Temperature and Humidity Resistance

**DOI:** 10.1002/advs.202301017

**Published:** 2023-04-29

**Authors:** Fanlin Tu, Zecong Ye, Yingxiao Mu, Xuwei Luo, Liyun Liao, Dehua Hu, Shaomin Ji, Zhiyong Yang, Zhenguo Chi, Yanping Huo

**Affiliations:** ^1^ School of Chemical Engineering and Light Industry Guangdong University of Technology Guangzhou 510006 China; ^2^ Key Laboratory of Polymeric Composite and Functional Materials of Ministry of Education School of Chemistry Sun Yat‐sen University Guangzhou 510275 China; ^3^ State Key Laboratory of Optoelectronic Materials and Technologies Sun Yat‐sen University Guangzhou 510275 China; ^4^ Analytical ＆ Testing Center Guangdong University of Technology Guangzhou 510006 China

**Keywords:** energy transfer, persistent luminescence, photoirradiation, polyimide, radical luminescence

## Abstract

Organic persistent luminescence (pL) systems with photoresponsive dynamic features have valuable applications in the fields of data encryption, anticounterfeiting, and bioimaging. Photoinduced radical luminescent materials have a unique luminous mechanism with the potential to achieve dynamic pL. It is extremely challenging to obtain radical pL under ambient conditions; on account of it, it is unstable in air. Herein, a new semialiphatic polyimide‐based polymer (A0) is developed, which can achieve dynamic pL through reversible conversion of radical under photoexcitation. A “joint–donor–spacer–acceptor” molecular design strategy is applied to effectively modulate the intramolecular charge‐transfer and charge‐transfer complex interactions, resulting in effective protection of the radical generated under photoirradiation. Meanwhile, polyimide‐based polymers of A1–A4 are obtained by doping different amine‐containing fluorescent dyes to modulate the dynamic afterglow color from green to red via the triplet to singlet Förster resonance energy‐transfer pathway. Notably, benefiting from the structural characteristics of the polyimide‐based polymer, A0–A4 have excellent processability, thermal stability, and mechanical properties and can be applied directly in extreme environments such as high temperatures and humidity.

## Graphical abstract

A simple and effective polymer structural design strategy is developed for semialiphatic polyimide systems with dynamic persistent luminescence features. The polyimide‐based matrix can stabilize the radical–triplet pair, which generates through the electron‐transfer process by photoirradiation, and simultaneously achieves excellent processability, thermal stability, and mechanical properties, and can be applied directly in extreme environments such as high temperatures and humidity.

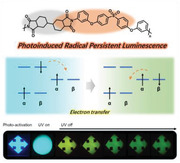

## 1 Introduction

The functional persistent luminescence (pL) material has developed rapidly in recent years,^[^ ^1^, ^2^, ^3^ ^]^ and has attracted much attention because of its potential applications in the fields of information dynamic encryption, advanced anticounterfeiting mechanism, and biomedicine.^[^ ^4^, ^5^, ^6^, ^7^ ^]^ Compared to inorganic materials, pure organic luminous materials are characterized by great processability, low cost, and biocompatibility.^[^ ^8^, ^9^, ^10^ ^]^ In order to obtain high‐performance organic pL materials, researchers have proposed several design strategies to suppress nonradiative transition through intermolecular interactions, such as crystal engineering, host–guest systems, carbon dots, and organic frameworks.^[^ ^11^, ^12^, ^13^, ^14^, ^15^ ^]^ As the research progresses, it will gradually advance toward industrialization, which places higher demands on the stability, mass production, and practical application of pL materials.^[^ ^16^ ^]^ Polymer pL material is an excellent candidate because of the flexibility, easy processing, and especially high thermal stability.^[^ ^17^ ^]^ Up to now, through various effective preparation strategies, including intrinsic polymer (main chain or branched chain) and embedding small molecules into polymer matrix, the polymer pL materials have made breakthrough progress.^[^ ^18^, ^19^, ^20^ ^]^ Noncovalent weak interactions within polymer molecules, such as hydrogen bonding, *π*–*π* stacking, and van der Waals forces, significantly weaken molecular vibrations and nonradiative deactivation, thus providing for efficient and long‐lifetime pL materials.^[^ ^21^, ^22^ ^]^ However, external factors such as high temperatures or humidity might easily increase the molecular motion in the polymer chains and thus disrupt the weak intermolecular interactions, which will seriously affect the luminescent properties of polymeric pL materials.^[^ ^23^ ^]^ Therefore, it is important to choose a suitable polymer model that can maintain pL performance in ordinary and extreme environments.

Polyimide (PI) is a kind of high‐performance polymeric material with outstanding thermal stability, chemical resistance, mechanical strength, and flexibility, which is widely utilized in the microelectronics and aerospace sectors.^[^ ^24^, ^25^, ^26^, ^27^ ^]^ Traditional PI is made by copolymerizing of aromatic dianhydride monomer with strong electron‐withdrawing ability and aromatic diamine monomer with high electron‐donating capacity.^[^ ^28^ ^]^ The main chain of aromatic PI is tightly packed, and there is high conjugation between aromatic rings, resulting in strong charge‐transfer complex (CTC) interactions.^[^ ^29^ ^]^ It leads to poor solubility and low optical transparency of aromatic PI, which affects their application in display field. In recent years, much research on the modification of classic aromatic PI has been conducted, mainly including the introduction of flexible structural groups, alicyclic segments, and isomeric structures.^[^ ^30^, ^31^, ^32^, ^33^, ^34^ ^]^ Among them, the development of semiaromatic PI is effective in breaking the strong conjugation between aromatic rings and regulating CTC strength. Under the premise of keeping extraordinary thermal and mechanical stability, the semiaromatic PI might improve the optical transmittance and obtain a certain luminous performance.^[^ ^35^ ^]^ At present, semiaromatic PI‐based photoelectric functional materials have been used in luminous films, electroluminescent devices, and sensors.^[^ ^36^, ^37^, ^38^, ^39^ ^]^ In addition, the structural characteristics of semiaromatic PI based on its rigid structure and intermolecular interaction will help to stabilize triplet excitons, making them promising to become new polymer pL materials with strong environmental adaptability.

**Figure 1:**
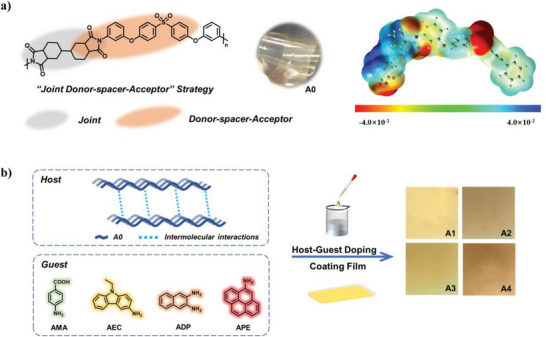
a) Design strategy, chemical structure, and the electrostatic potential distribution of A0. b) The structures of amine‐containing fluorescent dyes and schematic diagram of the preparation process.

Photoinduced radical luminescence (PIRL) materials are usually accompanied by dynamic luminescent phenomena such as luminescence enhancement, color change, or lifetime increase with the continuous irradiation of excitation light.^[^ ^40^ ^]^ Some PIRL materials have been reported presently, mainly including the crystalline and polymeric doping systems.^[^ ^41^, ^42^, ^43^, ^44^ ^]^ It is worth mentioning that the activation of PIRL requires two factors: the molecular structure conducive to the generation of radical and the matrix that could stabilize radical.^[^ ^45^ ^]^ The intrinsic polymer‐based PIRL materials with strong molecular structure designability offer considerable development potential. In this work, we designed and synthesized a novel semiaromatic PI (A0) based on the “joint–donor–spacer–acceptor” strategy in **Figure** **1**a, in which the “joint” and “spacer” groups could adjust the molecular skeleton conjugation, intramolecular charge‐transfer (ICT) and CTC interactions. The obtained freestanding film has excellent thermal stability, mechanical properties, and transparency. Surprisingly, the lifetime of A0 will be greatly increased under the continuous irradiation of excitation light, showing the dynamic pL phenomenon, accompanied by the enhancement of radical signal. As far as we know, this is the first case of PI‐based pL and intrinsic polymer‐based PIRL materials. In addition, polymer‐based doped luminophores (A1–A4) in Figure 1b obtained by microdoping (0.1% mol) with different amine‐containing fluorescent dyes could realize wide range color tuning of afterglow without affecting the physical properties of the films. More importantly, A0–A4 have remarkable high temperature and humidity resistance, that is, the stable pL phenomenon could still be observed at a high temperature of 100 °C or soaking in water. This novel PI‐based polymer with superior performance would become a competitive material in modern flexible display field.

## 2 Result and Discussion

### 2.1 Synthesis and Characterization

The semiaromatic PI (A0) was prepared by condensation and chemical imidization with commercialized bis[4‐(3‐aminophenoxy) phenyl] sulfone (BAPS‐M) and dicyclohexyl‐3,4,3″,4″‐tetracarboxylic dianhydride (HBPDA) based on the “joint–donor–spacer–acceptor” strategy, in which HBPDA as a “joint” unit can improve solubility and reduce rigidity due to the aliphatic ring structure. After the reaction, the diphenyl sulfone and anhydride in A0 are connected by diphenyl ether group to form a “donor–spacer–acceptor” unit, which inhibits ICT and CTC interactions. The electrostatic potential (ESP) distribution of model compound A0 was obtained by density functional theory (DFT) in Figure 1a, and the effective separation of positive and negative charges in the main chain was beneficial to enhance orientation polarization and reduce CTC effect. The synthetic routes of A0 are shown in Scheme S1 (Supporting Information). To confirm the chemical structure of A0, the Fourier transform infrared (FT‐IR) was preformed (Figure S1, Supporting Information). The infrared absorption bands at 1715 and 1776 cm^−1^, respectively, belonged to the symmetric and asymmetric stretching vibrations of C=O. The peak at 742 cm^−1^ represents the bending vibration of C=O in the imide ring. The stretching vibration peak of C—N in the imine ring appears at 1378 cm^−1^. A sharp peak appeared at 1151 cm^−1^ with a weaker absorption band at 1324 cm^−1^. These two bands are the characteristic S=O symmetric and asymmetric stretching of the sulfone group. The C—H stretching vibration peaks on saturated carbon and the C—H stretching vibration peaks on the benzene ring are displayed at 2862 and 2941 cm^−1^, respectively. After chemical imidization, the disappearance of the amide band at 1650 cm^−1^ together with the absence of carboxyl absorption peaks demonstrated complete imidization of the amide groups. The PI‐based polymer is highly soluble in polar solvents such as dimethylformamide (DMF), dimethylacetamide (DMAc), *N*‐methylpyrrolidone (NMP), and dimethyl sulfoxide (DMSO), and it could be cast in solution to form freestanding films. Film A0 presented light color and near‐transparent optical performance. The transmission UV–vis spectra observed in the visible region showed that the cutoff wavelengths were in the range of 348–353 nm with a high transparency of up to 83.5% at 450–700 nm in Figure S2 (Supporting Information). This is mainly due to the introduction of aliphatic rings and diphenyl ether group, which interrupted the conjugation and inhibited the generation of CTC.

Subsequently, a series of aromatic amine molecules of 4‐aminobenzoic acid (AMA),3‐amino‐9‐ethylcarbazole (AEC), 2,3‐diaminonapthalene (ADP), and 1‐amin‐opyrene (APE), which are selected as the dopants, were doped into film A0 with a doping concentration of 0.1% mol, and the prepared doped films were named as A1, A2, A3, and A4, respectively. Films A1–A4 with different energy levels produced colorful afterglow, assisted by the rigid PI‐based matrix and effective intermolecular interactions. The powder X‐ray diffraction (XRD) has been used to analyze the morphology of PI films A0–A4 in Figure S3 (Supporting Information). The results show broad dispersion peaks with the main peak 2*θ* in the range of 12°–25°, which revealed the amorphous structure and flexible film‐forming performance of all films. In addition, no distinct impurity peaks are appeared, indicating that guest dyes with microdoping can be uniformly dispersed in the matrix.

### 2.2 Thermal and Mechanical Properties

The thermal properties of film A0 were investigated by thermogravimetric analyses (TGA), differential scanning calorimetry (DSC), and dynamic mechanical analysis (DMA), and the results are shown in Figures S4–S6 and Table S1 (Supporting Information). Film A0 exhibited excellent thermal stabilities with an insignificant weight loss of up to 420 °C in atmospheres of nitrogen, and the 5% weight‐loss temperatures (*T*_d_) is nearly 429 °C. When the final combustion reaches 800 °C, 20% of their weight remains, which is carbonized residue after the combustion of aromatic compounds. Moreover, the glass transition temperature (*T*_g_) of A0 is about 210 °C, and it is also confirmed by DMA. It is slightly lower than aromatic PI due to the introduction of aliphatic ring structures and ether–oxygen bonds (—O—) in the A0 structure, which increases the flexibility of the main chain and weakens the intermolecular interactions.

The tensile strength measurements were performed to evaluate the mechanical properties of film A0. As presented in Figure S7 and Table S2 (Supporting Information), the tensile strength and modulus of film A0 are 119.09 MPa and 2.34 GPa, respectively, and the elongation at break is 15.06%, showing great mechanical features. The semirigid structure could ensure the superior tensile strength of film A0, and the high elongation at break profits from the inclusion of flexible groups in the molecular structure, which might reduce the stress in the tensile process. The significant thermal stability and mechanical properties of such a soluble semialiphatic PI are expected to meet the requirements of the high‐efficiency roll‐to‐roll process and high processing temperature in flexible display industry.

### 2.3 Photophysical Properties

**Figure 2:**
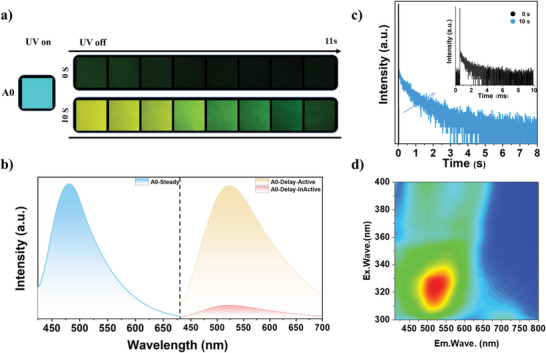
a) Photographs and b) steady‐state and transient photoluminescence (delayed 8 ms) spectra of film A0 taken under 365 nm UV light on and off after 0 and 10 s irradiation. c) Lifetime decay curve (blue line) of the emission band of A0 around 525 nm before and after photoactivation under ambient conditions. The inset shows a magnified plot (black line) of the A0 lifetime decay curve before photoactivation. d) Excitation–phosphorescence emission mapping of A0 after photoactivation.

In the film state, A0 appeared as the cyan emission at 480 nm with the photoluminescence quantum yields (PLQY) of 21% under a UV lamp in **Figure** **2**a,b. It is interesting that various pL could be observed by turning off the UV lamp after different irradiation time. It had no afterglow, and weak decay photoluminescence signals at 522 nm could be detected when UV light up and turn off immediately. By comparison, the luminance and duration of afterglow in film A0 were significantly improved after continuous UV light irradiation for 10 s. The lifetime of the emission of around 525 nm was improved from 1.0 to 456.3 ms, as shown in Figure 2c, while phosphorescence QY increased from 0.2% to 10.6%. Furthermore, the long‐lifetime pL of film A0 is dominated by yellow–green emission between 475 and 550 nm when the excitation wavelength is changed from 300 to 400 nm in the excitation–emission spectrum in Figure 2d. To verify the luminous characteristics, the steady‐state and transient photoluminescence spectra of film A0 from 77 to 300 K are measured in Figure S8 (Supporting Information). It can be seen that the lifetime of film A0 tends to increase observably as the temperature drops, which confirmed the phosphorescence properties. In addition, the delayed photoluminescence spectrum after photoactivation at room temperature almost completely overlaps with the steady‐state photoluminescence spectrum at 77 K, suggesting the origin of pL from discrete chromophores in this system.

**Figure 3:**
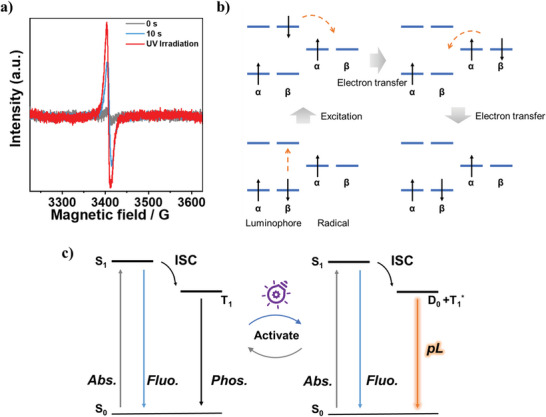
a) The electron paramagnetic resonance (EPR) spectra of film A0 after 0 and 10 s irradiation, and under UV light continuous irradiation. b) The electron transfer mechanism. c) Proposed mechanism of photoinduced radical pL phenomenon.

To uncover the mechanism, the electron paramagnetic resonance (EPR) tests of film A0 under different illumination conditions were tested (**Figure** **3**a), and there was no obvious radical signal under ambient conditions. After 10 s irradiation, the intensity of radical signal can be observed to increase greatly. The radical signal reached the maximum under continuous irradiation with the *g* value of 2.0034. In the open‐shell electron‐transfer mechanism (Figure 3b), the exciton on the luminophore *β* orbital could relax to the lower‐lying radical *β* orbital via intramolecular electron transfer after photoexcitation, and then spin back to the ground state through intramolecular electron transfer.^[^ ^46^ ^]^ Therefore, we propose a reasonable mechanism of the photoinduced radical pL phenomenon in Figure 3c. It is worth noting that the structures such as carbonyl groups, sulfone groups, ether bonds, and anhydrides facilitate intersystem crossing to obtain triplet excitons under photoexcitation. PI matrix has excellent oxygen barrier ability, and intermolecular interactions such as hydrogen bonds can effectively stabilize triplet excitons. Film A0 does not generate radicals when it is not illuminated, and only emits short‐lived phosphorescence. With the extension of UV lamp irradiation time, N or O atoms in A0 structure gradually transform into cationic radicals, thus enhancing the signal of radicals. Excitons on the radical *β* orbital would generate the radical–triplet pair with original electrons on the radical *α* orbital through the electron‐transfer process in the open‐shell electron‐transfer mechanism, and form the D_0_+T_1_^*^ energy level.^[^ ^47^ ^]^ Simultaneously, the protection of PI‐based matrix could stabilize the radical–triplet pair without quenching, and then obtain long‐lifetime pL. The film recovered to the initial state after stopping the excitation, indicating the reversible dynamic behavior between phosphorescence and radical emission realized.

**Figure 4:**
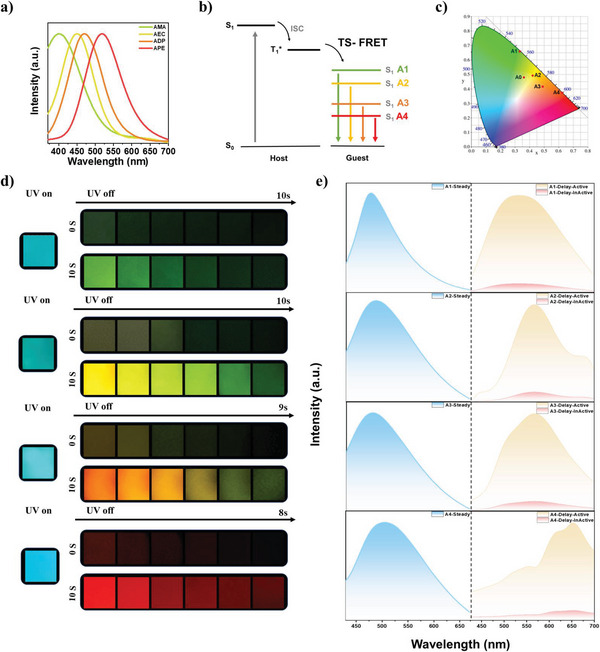
a) The steady‐state photoluminescence spectra of different amine‐containing fluorescent dyes. b) Schematic diagram of the triplet‐to‐singlet Förster‐resonance energy transfer (TS‐FRET) process based on the A1–A4 system. The energy level values of fluorescent dyes are calculated from the onsets of different emission peaks in the relative steady‐state photoluminescence spectra. c) The CIE coordinate diagram of A0–A4 afterglow emission. d) Photographs and e) steady‐state and transient photoluminescence (delayed 8 ms) spectra of films A1–A4 taken under 365 nm UV light on and off after 0 and 10 s irradiation.

Another challenge in the application of polymer‐based pL materials is to adjust the afterglow color. The material and time cost could be greatly saved by using the microdoping method without affecting the thermal or mechanical properties of pL materials. The triplet‐to‐singlet Förster‐resonance energy transfer (TS‐FRET) by doping pL molecules with the fluorescent dyes has gradually been widely applied in recent years.^[^ ^48^, ^49^, ^50^, ^51^ ^]^ Four kinds of amine‐containing guest dyes with different energy gaps were selected, and their photoluminescence spectra are shown in **Figure** **4**. As expected, the films A1–A4 realize multicolor pL in Figure 4d,e, and their photoactive performance is consistent with that of the host film A0, in which the main pL emission peaks are at around 540, 560, 620, and 650 nm, respectively. Meanwhile, the full width at half maximum (FWHM) of steady‐state photoluminescence spectra of films A1–A4 will increase with the energy level of guest dyes. The absorbance spectra of amine‐containing fluorescent dyes partially overlap the transient photoluminescence spectra of film A0 in Figure S9 (Supporting Information). Thus, the fluorescent dyes could accept the long‐lived triplet excitons from film A0 by the TS‐FRET process. The lifetime of films A1–A4 after photoactivation is reduced to 300–400 ms compared with A0 in Figure S10 and Table S3 (Supporting Information), which indicated that resonance excitation may transfer energy from PI matrix to guest dye. In order to understand the influence of trace doping on the properties of PI‐based polymer, we have carried out FT‐IR, transparency, thermal, and mechanical experiments on films A1–A4. As shown in Figure S1 (Supporting Information), the N—H bond of the guest dye has a weak tensile vibration peak at around 3300–3500 cm^−1^, and other vibration peaks are basically unchanged. The transmittance of films A1–A4 is slightly lower than that of the pure film in Figure S2 (Supporting Information), which might be due to the partial orientation of the guests in the films. The *T*_g_ and *T*_d_ of films A1–A4 are consistent with A0 (Figures S4–S6 and Table S1, Supporting Information), indicating that organic molecular doping has little effect on the thermal properties of PI films. In Figure S7 and Table S2 (Supporting Information), the tensile strength and elastic modulus of films A1–A4 will be affected by doping dyes, which may be a small amount of intermolecular forces that are destroyed after doping, but it can be seen from the numerical value that the mechanical properties of doped films are still at a high level. Overall, these results have important guiding significance on doping fluorescent dyes in intrinsic pL polymers by the microdoping method.

### 2.4 Applications

**Figure 5:**
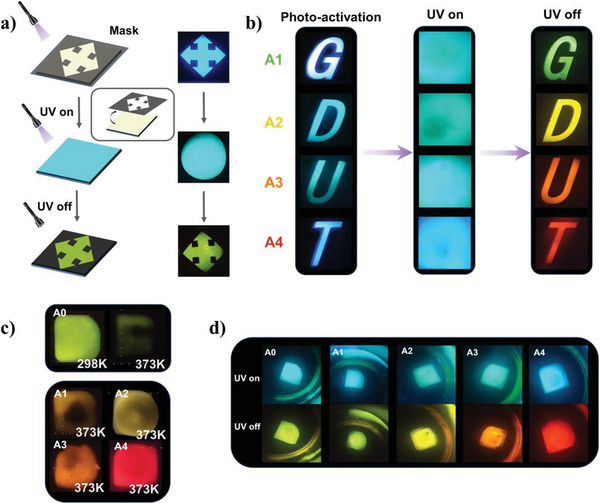
Schematic diagram of data encryption: illustration for the procedure of dynamic pL and the photographs of a) film A0 and b) films A1–A4. Afterglow images of films A0–A4 c) at high temperature and d) soaking in water for 48 h.

Based on the excellent dynamic pL response, we performed to explore the application of data encryption. As shown in **Figure** **5**a, film A0 is partially photoactivated against the mask and a distinct afterglow pattern can be observed. The character code “GDUT” was following assembled using films A1–A4, respectively. Under the excitation of the UV light, only the cyan emission is direct‐viewing without any information on the film in Figure 5b. Interestingly, the colorful “GDUT” pattern of films A1–A4 could be appeared clearly when closed the UV lamp. Thus, this special dynamic pL feature can easily and effectively carry out reversible multilevel data encryption and high‐density data storage. In addition, high temperature and water molecules in the environment will increase the nonradiative transition of luminous materials. Developing polymer‐based pL materials with high temperature and humidity resistance is beneficial to practical application, but the related research is still in the initial stage. To understand the high‐temperature resistance of materials, films A0–A4 were heated on a heating table, and the pL phenomenon could still be observed at a temperature as high as 100 °C in Figure 5c. This is due to the heat resistance and barrier of PI materials, which can effectively prevent the thermal quenching of triplet excitons. It is very rare for organic pL materials to continue to be used at such a high temperature. In terms of water resistance, we are surprised to discover that the photoactivated films A0–A4 after immersed in water for 48 h, and the pL performance is not affected, which shows that they have excellent humidity resistance (Figure 5d). The static contact angle and the water absorption test were used to explore the water absorption performance of films A0–A4 in Figure S11 and Table S4 (Supporting Information). It is illustrated that the contact angle of a water droplet on the surface is 88.5° with the water absorption rate of about 0.72%, showing the certain hydrophobicity. The introduction of aliphatic ring structure effectively strengthened the humidity resistance of A0. The contact angle of a water droplet on the surface and water absorption of the doped films are slightly lower than film A0, but the difference is not significant, indicating that the doping has little effect on the hydrophobicity of the system.

## 3 Conclusion

In summary, we have developed a highly efficient dynamic pL system for PI‐based polymers that could influence the luminous intensity and lifetime by modulating the transition of radical through continuous UV irradiation for the first time. By introducing the alicyclic and flexible structure into the main chain of PI, the ICT and CTC interactions could be effectively regulated, resulting in the synthesized PI‐based polymer with excellent luminescent and mechanical properties. By microdoping with different amine‐containing fluorescent dyes, the dynamic afterglow color of the doped films can be easily adjusted. Meanwhile, films A0–A4 still produce significant afterglow at a high temperature of 100 °C or soaking in water, showing superior resistance to high temperatures and humidity. These results provide an effective molecular design strategy for PI‐based polymeric materials with dynamic pL properties, and hold promise for applications in data security, advanced anticounterfeiting and displays in extreme environments.

## Conflict of Interest

The authors declare no conflict of interest.

## Supporting information

Supporting Information

## Data Availability

The data that support the findings of this study are available from the corresponding author upon reasonable request.
