# Supplementary material for: Photoinduced Radical Persistent Luminescence in Semialiphatic Polyimide System with Temperature and Humidity Resistance
Source: Adv Sci (Weinh). 2023 Apr 29;10(21):2301017. doi: 10.1002/advs.202301017 (PMC10375117; doi:10.1002/advs.202301017)
Supplement: Supplementary file 1 — Supporting Information [file ADVS-10-2301017-s001.pdf]

ADVANCED SCIENCE
Open Access

# Supporting Information

for *Adv. Sci.*, DOI 10.1002/advs.202301017

Photoinduced Radical Persistent Luminescence in Semialiphatic Polyimide System with Temperature and Humidity Resistance

*Fanlin Tu, Zecong Ye, Yingxiao Mu*, Xuwei Luo, Liyun Liao, Dehua Hu, Shaomin Ji, Zhiyong Yang, Zhenguo Chi* and Yanping Huo**

Supporting Information

**Photoinduced Radical Persistent Luminescence in Semi-Aliphatic Polyimide System with Temperature and Humidity Resistance**

*Fanlin Tu, Zecong Ye, Yingxiao Mu*, Xuwei Luo, Liyun Liao, Dehua Hu, Shaomin Ji, Zhiyong Yang, Zhenguo Chi* and Yanping Huo**

## 1. Experimental Section

**Materials:** Bis[4-(3-aminophenoxy) phenyl] sulfone (BAPS-M), dicyclohexyl-3,4,3',4'-tetracarboxylic dianhydride (HBPDA), 4-aminobenzoic acid ,3-amino-9-ethylcarbazole,2,3-diaminonapthalene, 1-amin-opyrene, pyridine, acetic anhydride were purchased from Bide Pharmatech Co. Ltd (Shanghai, China). All other reagents and solvents (analytical grade) were purchased from Bide Pharmatech Co. Ltd(Shanghai, China). All materials used without further purification.

**Characterizations:** Fourier transform infrared spectra was measured by a FT-IR spectrophotometer (Thermo Scientific Nicolet 6700, USA) by attenuating total reflection from 400 $cm^{-1}$ to 4000 $cm^{-1}$. Thermal degradation was recorded using a thermogravimetric analyzer (TGA, SDTQ600, Germany) from room temperature to 800 °C at a heating rate of 10 °C/min in nitrogen.The X-ray powder diffractometer (XRD) spectrum was measured by Ultima III, Rigaku Corporation.Use Shimadzu ultraviolet visible spectrophotometer UV-2700 to test the light transmittance of the sample.The photoluminescence spectra were collected with a spectrometer from Steady state transient fluorescence spectrometer FLS980.Use optical contact angle measuring instrument OCA40 to measure the hydrophobicity of the sample. The stress-strain curves were measured by electronic universal testing machine (Inspekt Table Blue 5KN,

Germany, Hegewald & Peschke). Molecular weight was tested by gel permeation chromatography (Alliance e2695 Separation Molecule/2414 RI Detector, USA, Waters).

**Synthesis of A0:** At room temperature, add 4,4 '- ((sulfonylbis (4,1-phenylene)) bis (oxy)) diphenylamine (4.325g) and N, N-dimethylacetamide (23.5ml) into a flask with mechanical agitation and nitrogen protection, and stir until the solution is clear. Add dicyclohexyl-3,4,3',4'-tetracarboxylic dianhydride (3.063g) slowly and in batches under the condition of vigorous stirring, and add it in three times. The amount of the last addition is less than or equal to half of the previous one, and react for 8h at room temperature. Add 5ml acetic anhydride and 2.5ml pyridine, raise the temperature to 140 ℃ at the same time, and react for 12h under solvent reflux. The obtained polyimide solution is subjected to precipitation and suction filtration in a mixed solution of methanol and deionized water (volume ratio is 4:1) with a volume of 8 times. The solid obtained after suction filtration is dissolved in N, N-dimethylacetamide. The polyimide solution is subjected to repeated precipitation and suction filtration treatment for 3 times. It is dried under vacuum at 80 ℃ for 12h to obtain gray white polyimide powder. The average molecular weight (Mn) of the polyimide powder obtained is about 26500, which is relatively stable, and the polydispersity (PDI) is about 1.2.

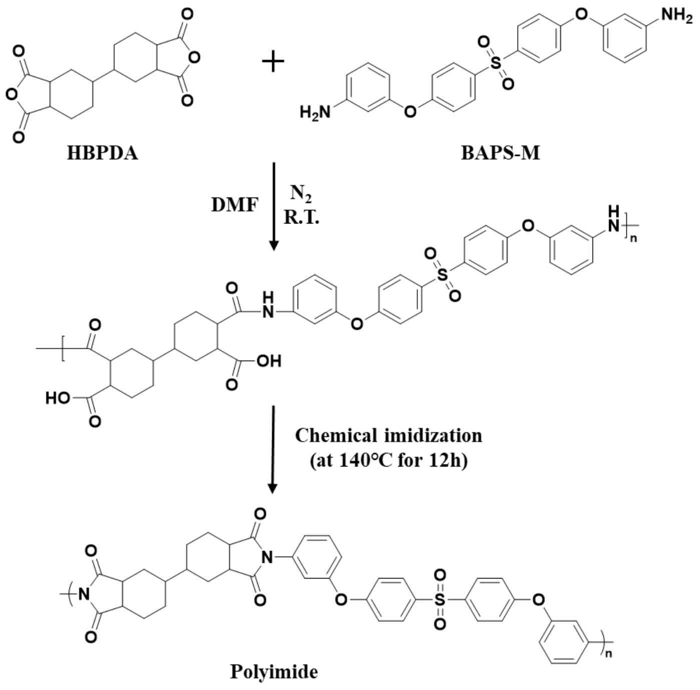


**Scheme S1.** The synthetic routes of A0.

**Synthesis of A1:** Powdery polyimide was dissolved in DMAc at a solid content of 25 wt %, the organic molecules are then added and stirred until complete dissolution. The PI/organic molecule mixture (volume ratio of 40:1) was dropped on a glass plate, and the temperature was stepped up to 170 °C to remove the DMAc residue to prepare a doped polyimide film.

**Synthesis of A2, A3 and A4:** The procedure was analogous to that described for A1.

## 2. Supplementary Figures and Tables

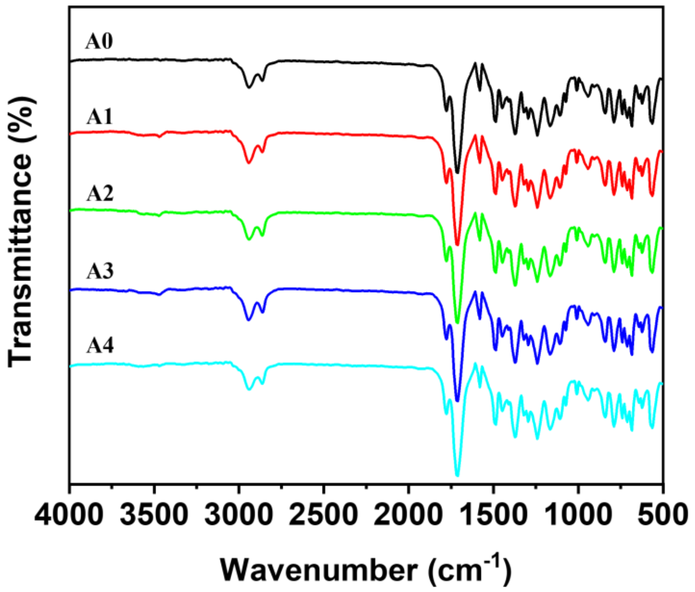


**Figure S1.** Fourier transform infrared (FT-IR) spectra of A0-A4.

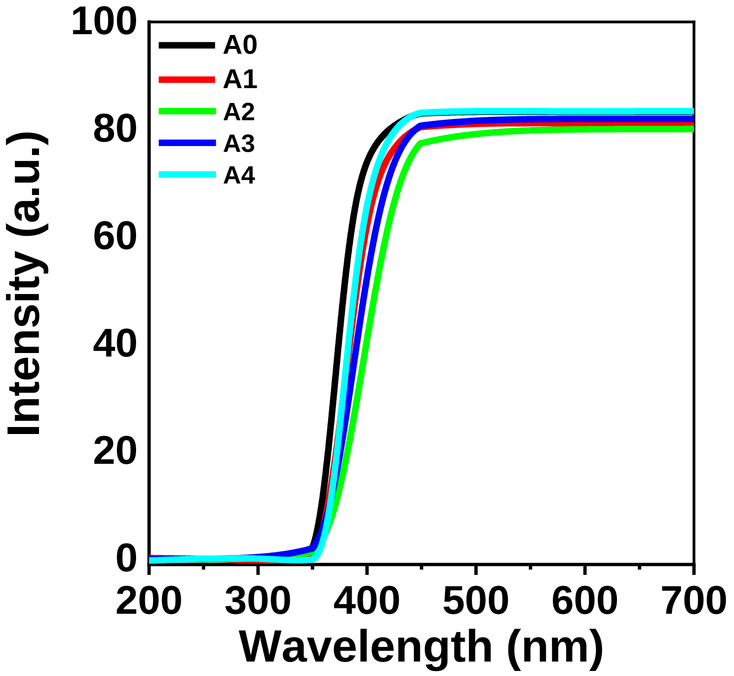


**Figure S2.** UV-Vis transmission spectra of A0-A4.

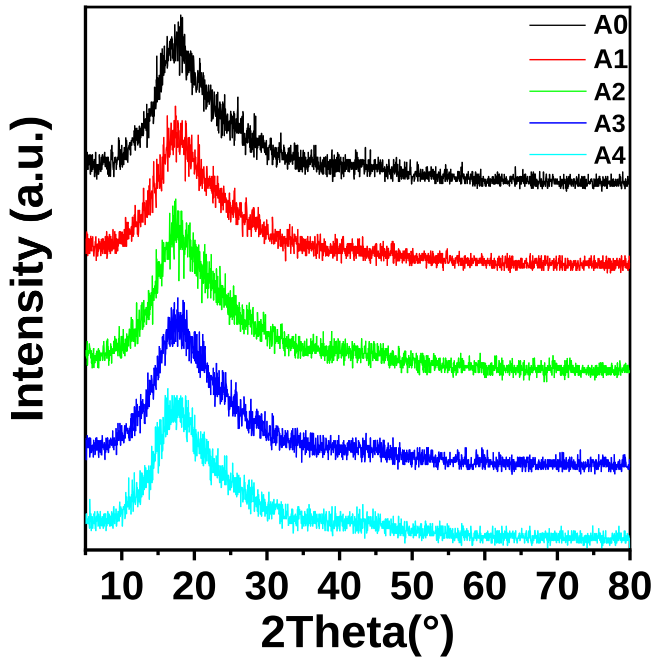


**Figure S3.** XRD pattern of A0-A4.

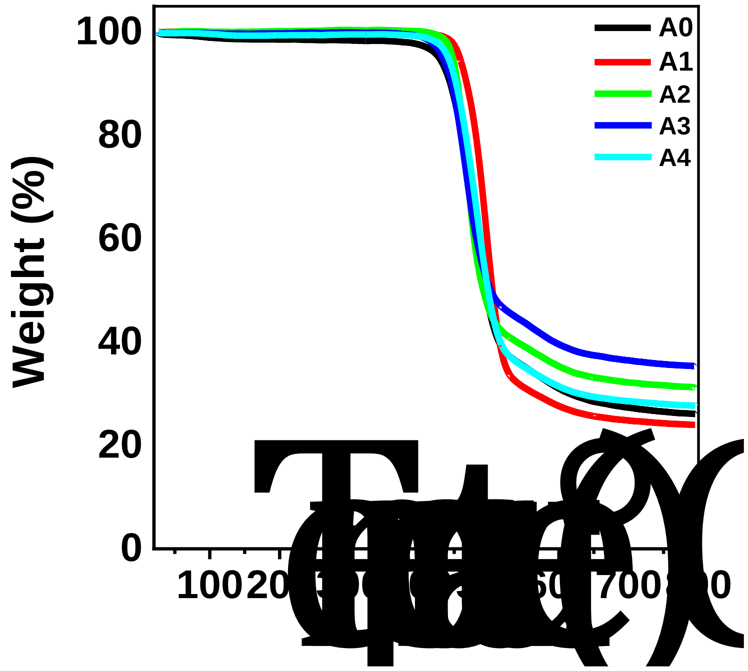


**Figure S4.** TGA curves of A0-A4.

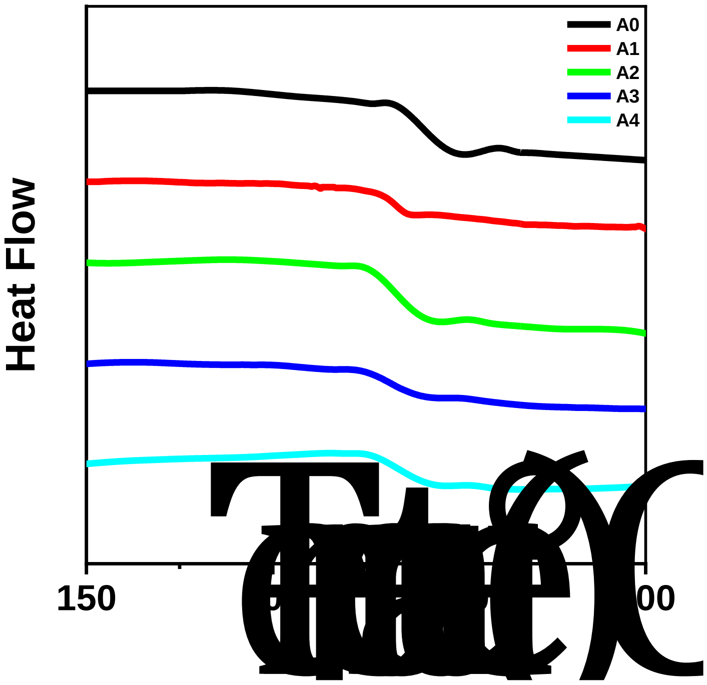


**Figure S5.** DSC curves of A0-A4.

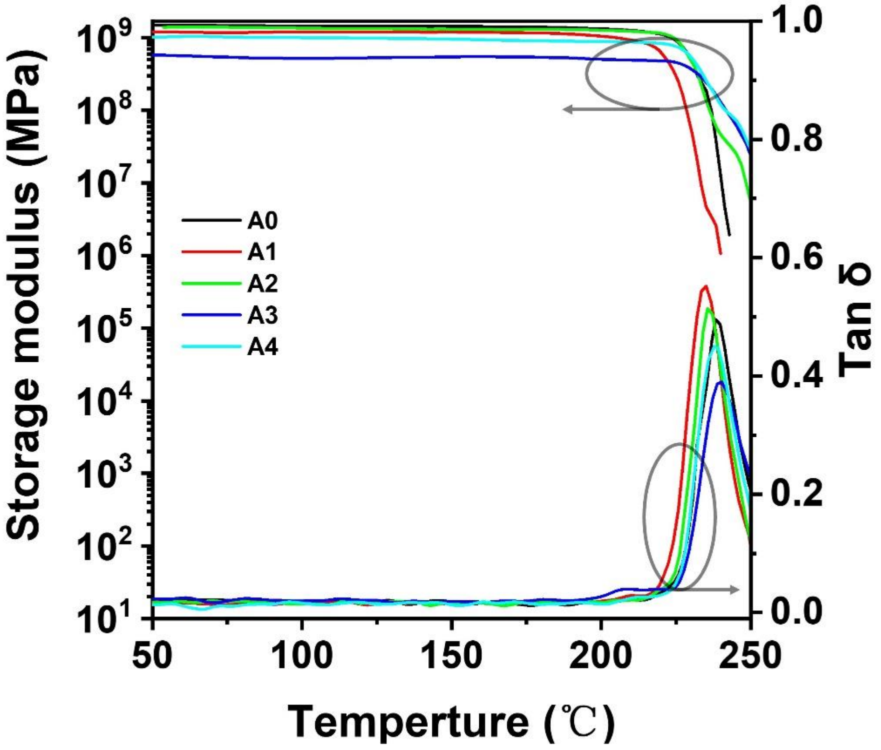


**Figure S6.** DMA curves of A0-A4.

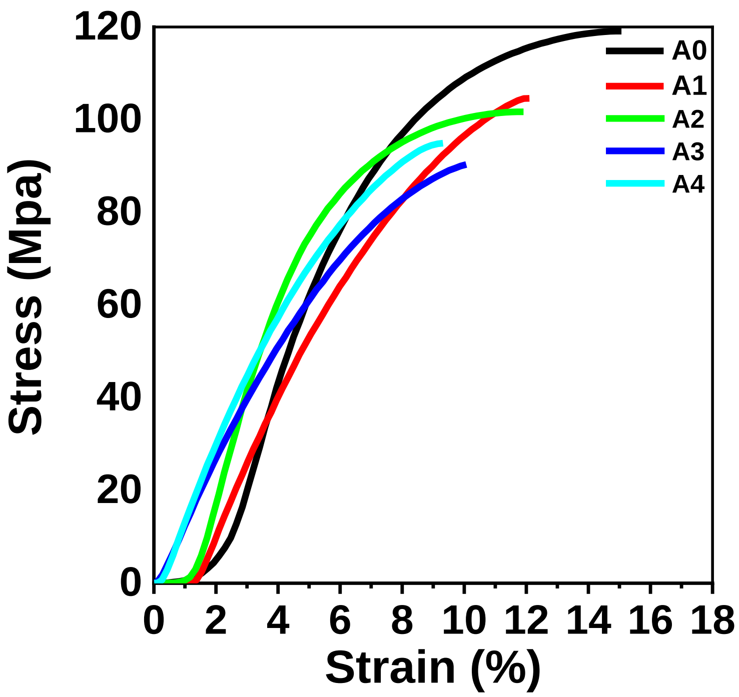


**Figure S7.** Stress-strain curves of A0-A4.

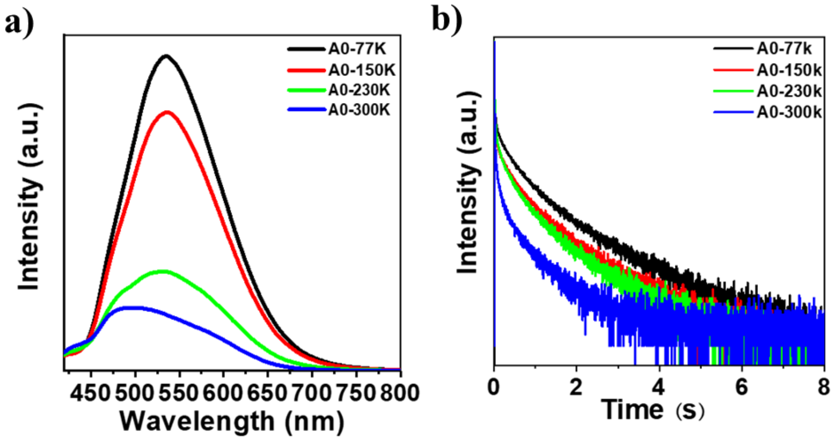


**Figure S8.** a) Steady-state photoluminescence spectra and b) lifetime decay curve of film A0 at different temperatures.

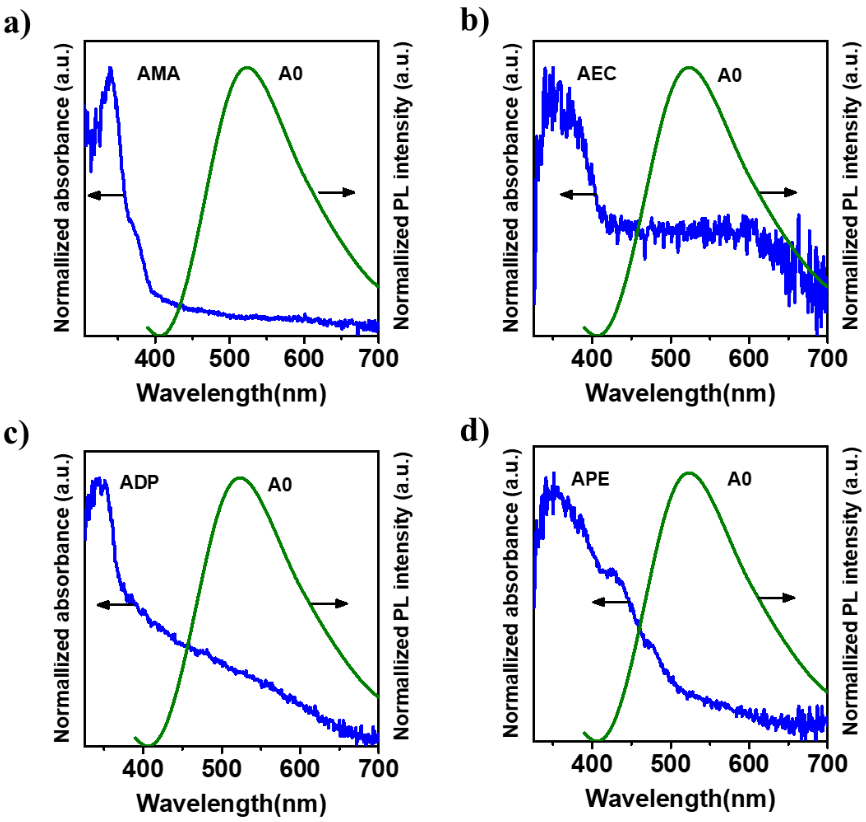


**Figure S9.** Spectral overlap between the emission (delayed 8 ms, green line) of film A0 and the solid-state absorbance (blue line) of amine-containing fluorescent dyes.

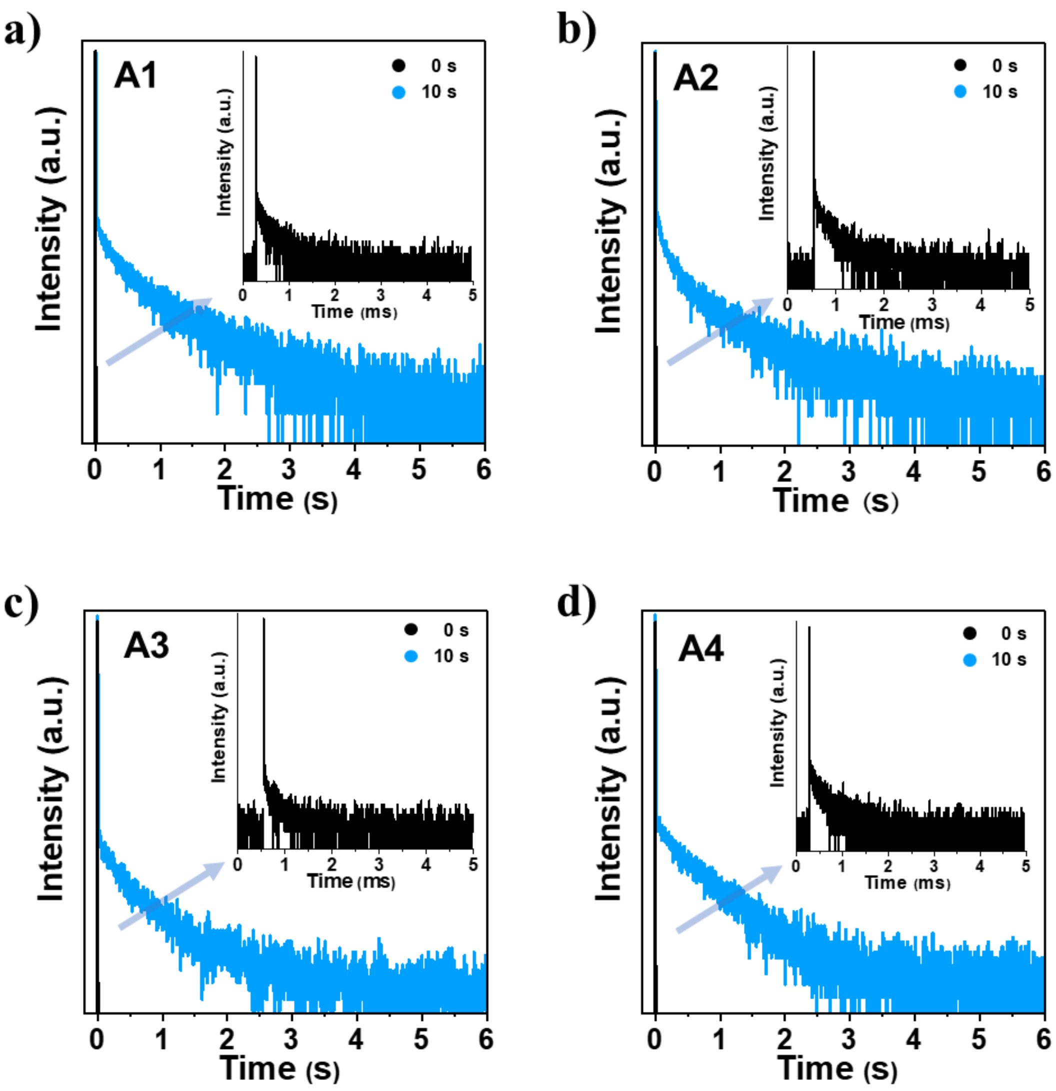


**Figure S10.** Lifetime decay curve (blue line) of the emission band of a) A1 around 540 nm, b) A2 around 560 nm, c) A3 around 620 nm and d) A4 around 650 nm before and after photo-activation under ambient conditions. The inset showed a magnified plot (black line) of the relevant lifetime decay curve before photo-activation.

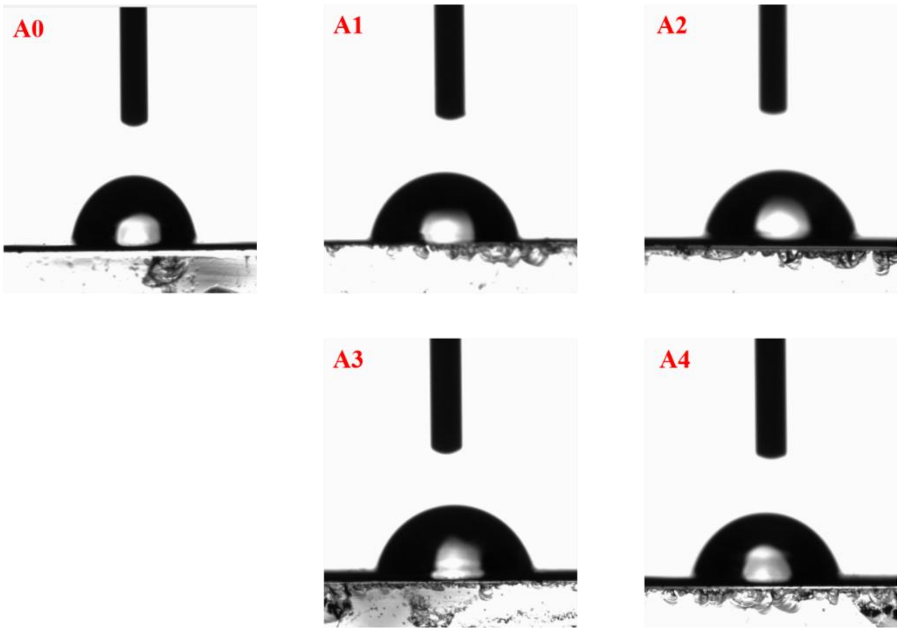


**Figure S11.** The water contact angle photographs of A0-A4.

**Table S1.** Summary of thermal performance data of A0-A4.

| | A0 | A1 | A2 | A3 | A4 |
|---|---|---|---|---|---|
| $T_g$ (℃) | 210 | 206 | 211 | 203 | 208 |
| 5% $T_d$ (℃) | 429 | 458 | 448 | 434 | 442 |

**Table S2.** Summary of mechanical performance data of A0-A4.

| | A0 | A1 | A2 | A3 | A4 |
|---|---|---|---|---|---|
| Tensile Strength (MPa) | 119.09 | 104.61 | 101.70 | 90.32 | 94.93 |
| Young's modulus (GPa) | 2.34 | 1.58 | 1.67 | 1.53 | 1.72 |
| Elongation break (%) | 15.06 | 12.10 | 11.91 | 10.07 | 9.31 |

**Table S3.** The lifetime value of A0-A4 before and after photo-activation.

| | A0 | A1 | A2 | A3 | A4 |
|---|---|---|---|---|---|
| 0 s | 981.87 µm | 157.74µm | 189.45µm | 178.70µm | 199.01µm |
| 10 s | 456.30ms | 384.54ms | 349.79ms | 334.98ms | 367.70ms |

**Table S4.** Water contacts angle and water absorption of A0-A4.

| | A0 | A1 | A2 | A3 | A4 |
|---|---|---|---|---|---|
| Water contacts angle (°) | 88.5 | 79.9 | 82.5 | 74.6 | 80.7 |
| Water absorption (%) | 0.72 | 0.85 | 0.83 | 0.92 | 0.88 |
